# Unveiling social distancing mechanisms via a fish-robot hybrid interaction

**DOI:** 10.1007/s00422-021-00867-9

**Published:** 2021-03-17

**Authors:** Donato Romano, Cesare Stefanini

**Affiliations:** 1 The BioRobotics Institute, Sant’Anna School of Advanced Studies, Viale Rinaldo Piaggio 34, 56025 Pontedera, Pisa, Italy; 2 Department of Excellence in Robotics and AI, Sant’Anna School of Advanced Studies, 56127 Pisa, Italy; 3 Healthcare Engineering Innovation Center (HEIC), Khalifa University, Abu Dhabi, UAE

**Keywords:** Animal–robot interaction, Ethorobotics, Collective behaviour, Pathogen transmission, Social distancing

## Abstract

Pathogen transmission is a major limit of social species. Social distancing, a behavioural-based response to diseases, has been regularly reported in nature. However, the identification of distinctive stimuli associated with an infectious disease represents a challenging task for host species, whose cognitive mechanisms are still poorly understood. Herein, the social fish *Paracheirodon innesi*, was selected as model organism to investigate animal abilities in exploiting visual information to identify and promote social distancing towards potentially infected conspecifics. To address this, a robotic fish replica mimicking a healthy *P. innesi* subject, and another mimicking *P. innesi* with morphological and/or locomotion anomalies were developed. *P. innesi* individuals were attracted by the healthy fish replica, while they avoided the fish replica with morphological abnormalities, as well as the fish replica with an intact appearance, but performing locomotion anomalies (both symptoms associated with a microsporidian parasite infesting *P. innesi* and other fish). Furthermore, the fish replica presenting both morphology and locomotion anomalies in conjunction, triggered a significantly stronger social distancing response. This confirms the hypothesis that group living animals overgeneralize cues that can be related with a disease to minimize transmission, and highlights the important role of visual cues in infection risk contexts. This study prompts more attention on the role of behavioural-based strategies to avoid pathogen/parasite diffusion, and can be used to optimize computational approaches to model disease dynamics.

## Introduction

The complex biological mechanisms regulating social behaviour are still poorly understood, although this phenomenon is widespread in the animal kingdom, including humans (Adolphs 2003; Krause and Ruxton 2002; Morrell and James 2008). Several evolutionary hypothesis have been proposed to explain animal aggregations (Allee 1927), including boosted fluid dynamics during locomotion (Barber and Folstad 2000), increased probability of mate encounters (Agrillo et al. 2008), reduced risk of predation (Parrish et al. 1989). However, group living may also presents some drawback such as the increased cost of competition (Thünken et al. 2014). A major cost of animal aggregations is the facilitation of pathogen transmission (Dobson 1988; Manlove et al. 2014). For easily transmitted pathogens (i.e. aerosolized transmission routes), the infection rate tends to increase with increasing population density (Begon et al. 2002), while for pathogens requiring more intense contact for transmission (i.e. sexual transmission routes), the forces of infection are independent of population density.

Social distancing, based on the reduction of contact rates among individuals through behavioural changes, is an important aspect in reducing the transmission of a large number of diseases in humans (Reluga 2010). Studies on agent-based influenza simulations clearly indicate how slight behavioural changes produce important effects on transmission dynamics during an epidemic (Kelso et al. 2009).

Social distancing to limit the transmission of diseases has been documented across the Animal Kingdom, including lobsters, ants, non-human primates, Trinidadian guppies, etc. (Behringer et al. 2006; Stroeymeyt et al. 2018; Freeland 1976; Stephenson et al. 2018; Romano et al. 2020d; Townsend et al. 2020). Adaptive behaviour to environmental changes, is an important feature that is early developed in life by animals (Groneberg et al. 2020). Furthermore, social experiences during early stages of life have been proved to have prolonged consequences on social and other behaviours (Harlow et al. 1965; Shams et al. 2018; Groneberg et al. 2020). However, cognitive mechanisms, activated by social experience, that shape the decision-making process related to social distancing is an aspect that remains unexplored.

A reason justifying social distancing in group living animals can be found in the optimal spatial distribution within a group of animals during collective movements (Herbert-Read 2016). In this context, vision and mechanosensation were proved to play a key role to mediate avoidance reactions in social fish (Katz et al. 2011; Hein et al. 2018; Groneberg et al. 2020; Dreosti and López-Schier 2020). In particular, social distancing is an innate behaviour that is reinforced by early life experience, and it affects neuronal circuits producing long-term modifications in social interactions.

Herein, we investigated if animals can exploit visual information to identify conspecifics with superficial (visible) infections, and how this experience promotes social distancing behaviour.

The neon tetra *Paracheirodon innesi*, Myers (Characiformes: Characidae), one of the most popular social ornamental fish species (Chapman et al. 1998), has been used as model organism.

To carry out highly controllable experiments, and to avoid the spread of infectious diseases in the fish colony, a robotic fish replica mimicking a healthy neon tetra, and another one mimicking a neon tetra with morphological and locomotion anomalies associated with several diseases (Michel et al. 2002; Palacios et al. 2015; Langenmayer et al. 2015), were developed to interact with living *P. innesi*. This bionic interactive paradigm is based on the animal-robot interaction technology and ethorobotics, that provide innovative methodologies to study social interactions in animals, through the use of animal-like agents (Krause et al. 2011; Romano et al. 2019a, b). This biohybrid approach ensures highly standardized cues and experimental conditions, full control of the robotic agents in space and time, as well as enables to produce robotic cues resembling focal live subjects, or robotic cues having a stark contrast with them (Polverino and Porfiri 2013; Bierbach et al. 2020; Romano et al. 2020a, b, c).

The fish-robot social interaction presented in this study could contribute to further understand the mechanisms involved in the cognition of social species and the evolution of social distancing.

## Materials and methods

### Ethics statement

The present study complies with the Guidelines for the Use of Animals in Research (ASAB/ABS 2014), as well as to the legal requirements of Italian (D.M. 116192), and EU regulation (European Commission 2007). All experiments are behavioural tests, and no specific consents are needed in the country where the experiments were conducted.

### Animals rearing and general observations

*Paracheirodon innesi* individuals were purchased from an aquarium store in Pontedera (Pisa, Italy), and kept in 100 L aquaria filled with activated charcoal-filtered water under laboratory conditions at 25 ± 1 °C, and with a 16:8 h light: dark photoperiod. An air diffuser constantly aerated cultures, and water was completely replaced every seventh day. A commercial food (Tetramin® flake food) was used as fish diet, and was provided twice a day ad libitum. During experiments, the same aforementioned controlled conditions were maintained.

To illuminate the laboratory, overhead fluorescent daylight tubes (Philips 30 W/33) were used, and reflection and phototaxis were reduce by using diffused laboratory lighting. After each replicate, test tanks and the robotic fish were narrowly washed (Romano et al. 2017) to avert effects produced by olfactory cues from previous tests.

### Fish replicas design and robotic apparatus

**Fig. 1:**
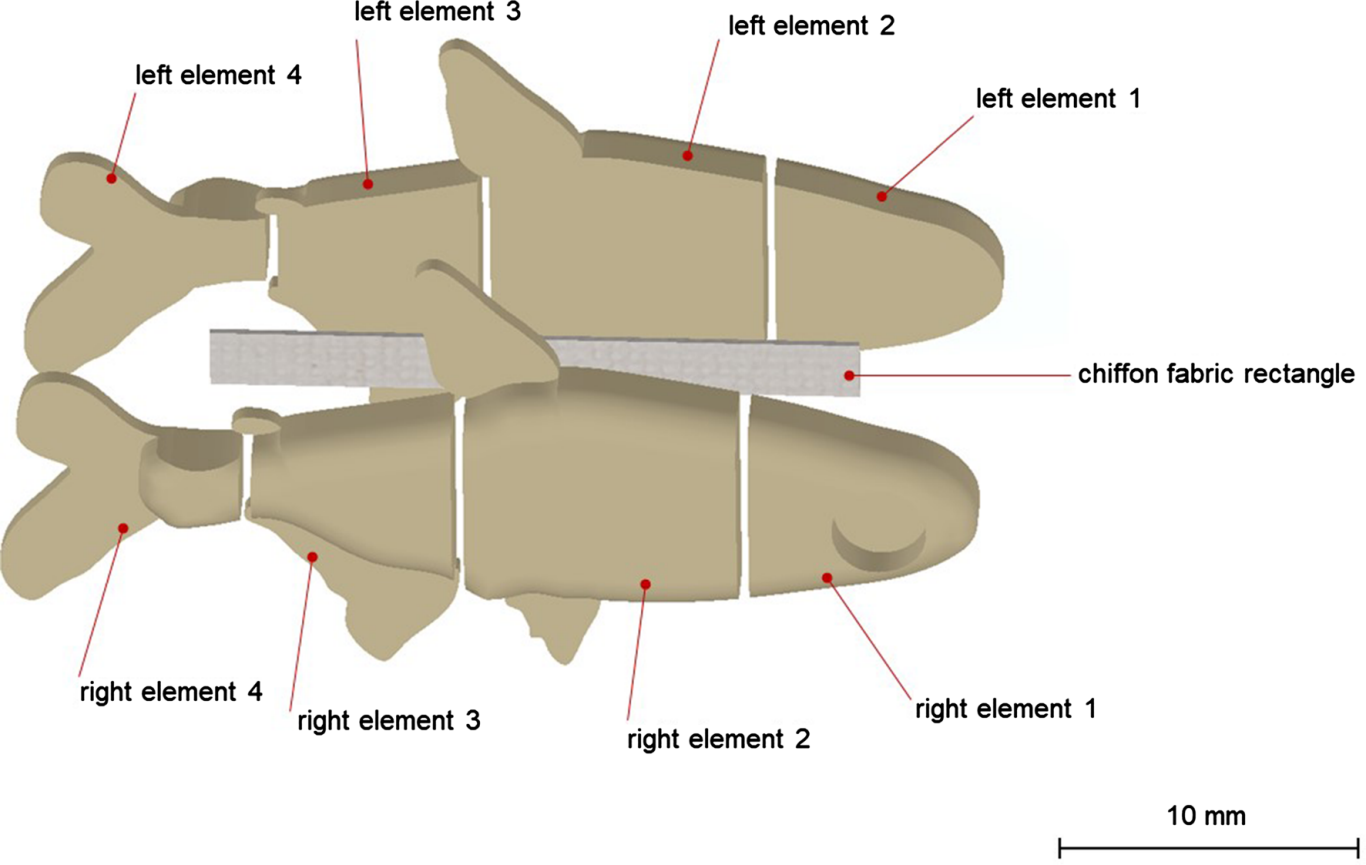
Schematic illustration of the fish replica design, showing the eight complementary elements, and the chiffon fabric rectangle on its sagittal plane

Fish replicas morphology was inspired by *P. innesi* adult individuals. Four pairs of elements were designed in SolidWorks (Dassault Systemes, Velizy-Villacoublay, France), fabricated in acrylonitrile butadiene styrene (ABS) by fast prototyping, and finally assembled by placing a chiffon fabric rectangle (18 × 3 mm) as sagittal plane between complementary elements (Fig. 1).

Once assembled, each fish replica was 27 mm long, 11 mm tall, and 4 mm wide, and presented a dorsal fin, a second dorsal fin, an anal fin, a caudal fin, two pelvic fins, and two ocular regions.

**Fig. 2:**
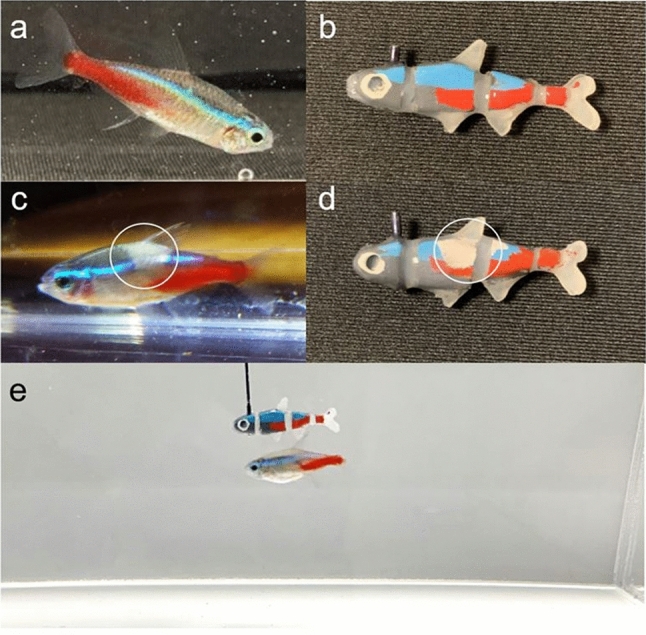
Snapshots indicating a *Paracheirodon innesi* healthy individual (**a**) and its mimicking fish replica (**b**); a *P. innesi* sick individual (**c**) and its mimicking fish replica (**d**); a focal *P.innesi* individual schooling with the healthy fish replica (**e**)

Non-toxic pigments were used to paint the fish replicas similarly to the colour pattern of *P. innesi* (Fig. 2a, b).

In the case of the fish replica mimicking a sick *P. innesi*, decoloured areas were left on its body to reproduce symptoms associated with *Pleistophora hyphessobryconis* (Michel et al. 2002; Novotný and Dvořák 2006) (Fig. 2c, d), a quite common Microsporidian parasite of neon tetras and other fish. A colorimeter (Nix Pro 2 Color Sensor) was used to record colour measurements (standard CIELab colour space coordinates) of both fish replicas (Table 1). Both fish replicas were covered by a thin layer of transparent silicone rubber (Dragon Skin), that along with their compliant body, increased the biomimetic appearance of fish replicas.

**Table 1:** Colour measurements of the biomimetic colour pattern of the fish replicas ± standard error

	L*	a*	b*
Fish replica's blue area	59.8 ± 0.26	−24.1 ± 0.16	−25.1 ± 0.14
Fish replica's red area	44.84 ± 0.44	52.12 ± 0.21	34.12 ± 1.02
Fish replica's decoloured area	86.4 ± 0.05	4.46 ± 0.02	6.06 ± 0.07

L* represent the lightness component, a* (from red to green) and b* (from blue to yellow) are the two chromatic components

A trajectory generator located above the test tank was used to move the fish replicas. According to the experimental context, the healthy fish replica and the sick fish replica were connected to the trajectory generator through a rod (⌀ 0.5 mm), or through a nylon wire (⌀ 0.5 mm). The nylon wire determined an unstable position of the fish replicas’ body, when moved by the trajectory generator, staging a swimming difficulty, a more severe symptom caused by *P. hyphessobryconis* (Michel et al. 2002; Novotný and Dvořák 2006). The trajectory generator had two stepper motors, actuating two sliding axis (i.e. x and y axes), and controlled by a microcontroller. It operated on an area of around 400 × 200 mm (accuracy of the path following = 0.01 mm). Plotted trajectories were converted in G-Code (i.e. RS-274), and subsequently sent to the microcontroller. The microcontroller was connected to an external processor that managed the plotting and code conversion phases.

### Animal-robot behavioural experiment

**Fig. 3:**
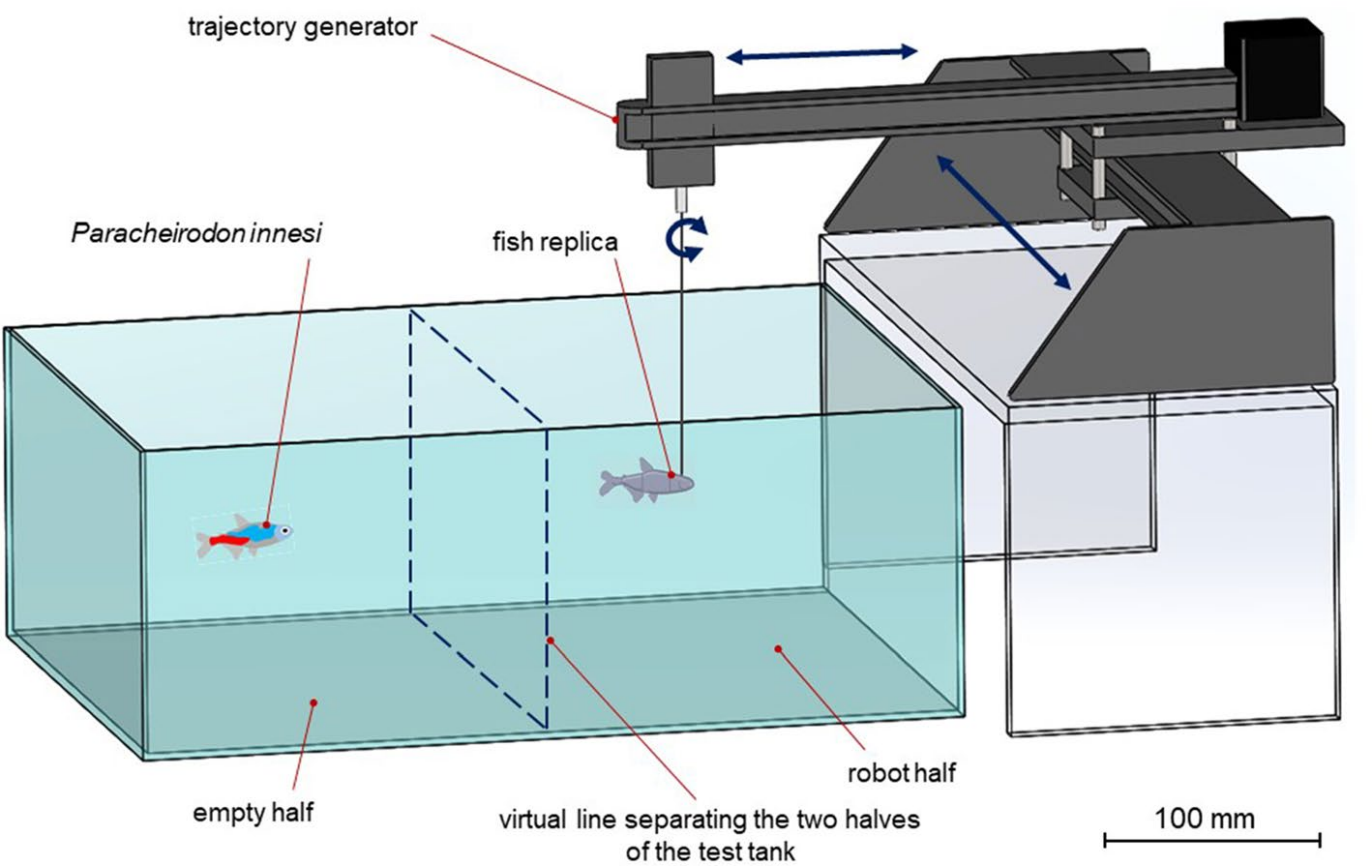
Experimental apparatus for the animal-robot behavioural experiments. *P. innesi* and the fish replica are not drawn to scale

*Paracheirodon innesi* were individually transferred in a test tank (400 × 300 × 150 mm; length × width × depth), virtually divided in 2 halves of equal size: an empty half and a robot half. Before the beginning of a test, a neon tetra was placed in the empty half that was separated by the robot half by an opaque partition. The test started when, after an acclimatation phase of five minutes, the opaque partition was removed showing and enabling the interaction with the fish replica in the robot half (Figs. 2e, and 3). The trajectory generator moved the fish replica with a velocity of 5 mm/s, on a circular trajectory (⌀ 100 mm) in the robot half of the test tank. The test lasted 20 min. To avoid orientation biases, the test tank was rotated at the end of each replicate.

Fish were exposed to 4 contexts: (i) healthy fish replica (e.g. fish replica with a colour pattern reasonably similar to *P. innesi*, and connected to the trajectory generator through the rod); (ii) sick fish replica (e.g. fish replica with decoloured areas on its body, and connected to the trajectory generator through the rod); (iii) healthy fish replica with locomotion anomalies (e.g. fish replica with a colour pattern close to *P. innesi*, and connected to the trajectory generator through the nylon wire); (iv) sick fish replica with locomotion anomalies (e.g. fish replica with decoloured areas on its body, and connected to the trajectory generator through the nylon wire).

The time spent by *P. innesi* in the 2 halves of the test tank, as well as the duration of the schooling behaviour (e.g. moving collectively with other conspecifics at a distance of at least 5 body lengths from each other [O'Steen et al. 2002]) of *P. innesi* towards the fish replicas, was recorded. Fish individuals were tested only once. For each context, 20 fishes were analyzed.

### Statistical analyses

Data on the impact of the 4 contexts on the time spent by *P. innesi* individuals in the 2 halves of the test tank showed a nonparametric distribution (Shapiro–Wilk test, goodness of fit *P* < 0.05), therefore they were analysed by using nonparametric statistics, and in particular the Wilcoxon test (*P* = 0.05). Furthermore, data on the time spent in the empty half, the robot half, as well as the schooling behaviour duration in *P. innesi* individuals postexposure to different contexts, were also not normally distributed (Shapiro–Wilk test, goodness of fit *P* < 0.05). So, here Kruskal–Wallis test followed by Steel–Dwass test (*P* = 0.05) were performed. R software v3.6.1 (Stats Package), was used to analyse the data.

## Results

**Fig. 4:**
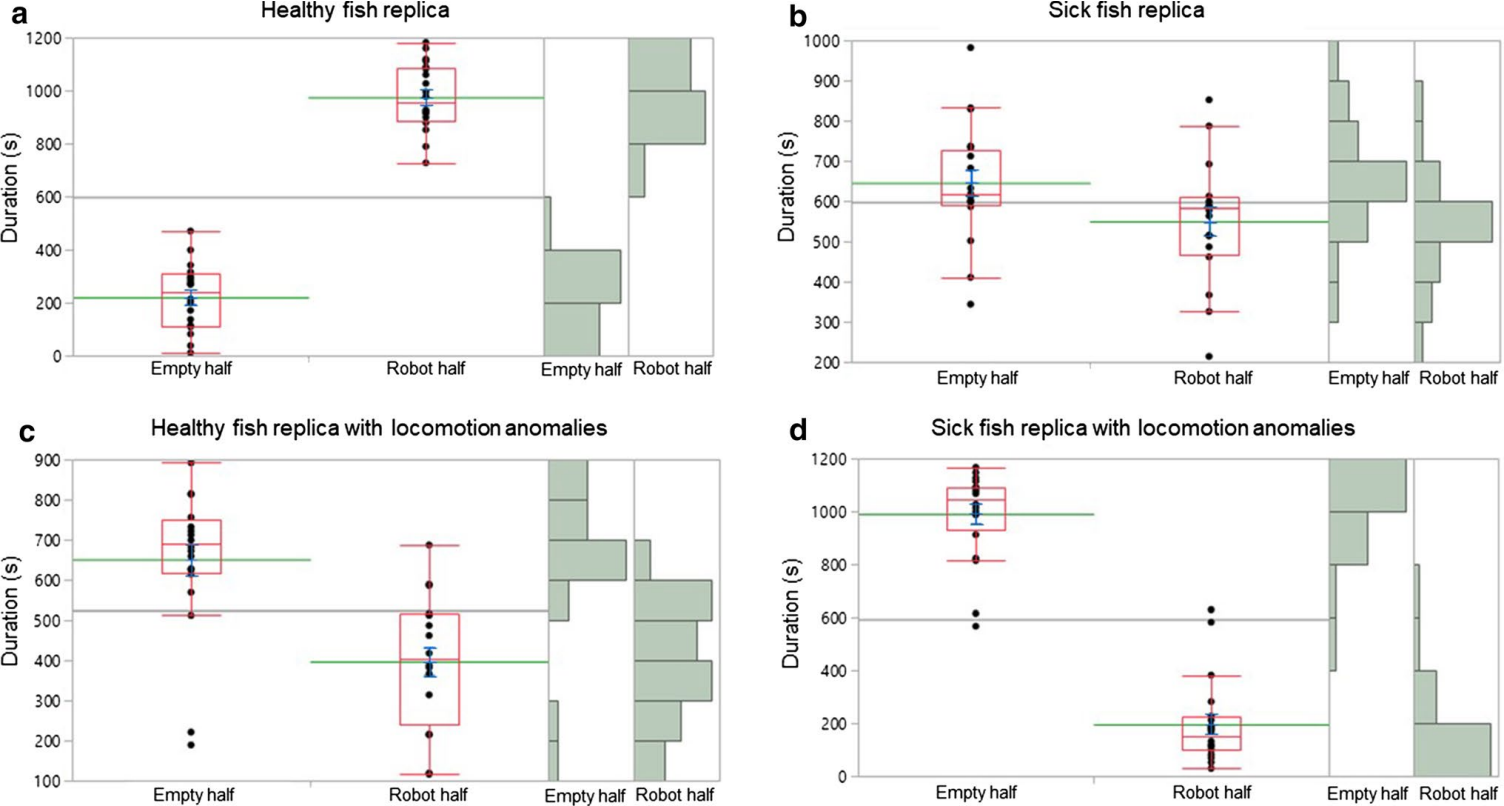
The preference of *Paracheirodon innesi* subjects to swim in the empty half or in the robot half of the test tank is affected by **a** the healthy fish replica, **b** the sick fish replica, **c** the healthy fish replica with locomotion anomalies, and **d** the sick fish replica with locomotion anomalies (Wilcoxon test, *P* > 0.05). The red line present in each box plot indicates the median and its range of dispersion (lower and upper quartiles, as well as outliers). The green line indicates the mean, and the blue T-bars show standard error value. For each box plot, on the right, data distribution is shown on histograms

Fish individuals spent a significantly longer time in the robot half compared to the empty half (χ2 = 29.2; *d.f.* = 1; *P* < 0*.*0001) when the healthy fish replica was presented (Fig. 4a). Conversely, when the sick fish replica was presented, fish spent a significantly shorter time in the robot half (*χ*2 = 5.6; *d.f.* = 1; *P* = 0*.*0179) (Fig. 4b). When the healthy fish replica with locomotion anomalies was presented, fish spent a significantly shorter time in the robot half (*χ*2 = 17; *d.f.* = 1; *P* < 0*.*0001) (Fig. 4c). When the sick fish replica with locomotion anomalies was presented, fish spent a significantly shorter time in the robot half compared to the empty half (*χ*2 = 28.4; *d.f.* = 1; *P* < 0*.*0001) (Fig. 4d).

**Fig. 5:**
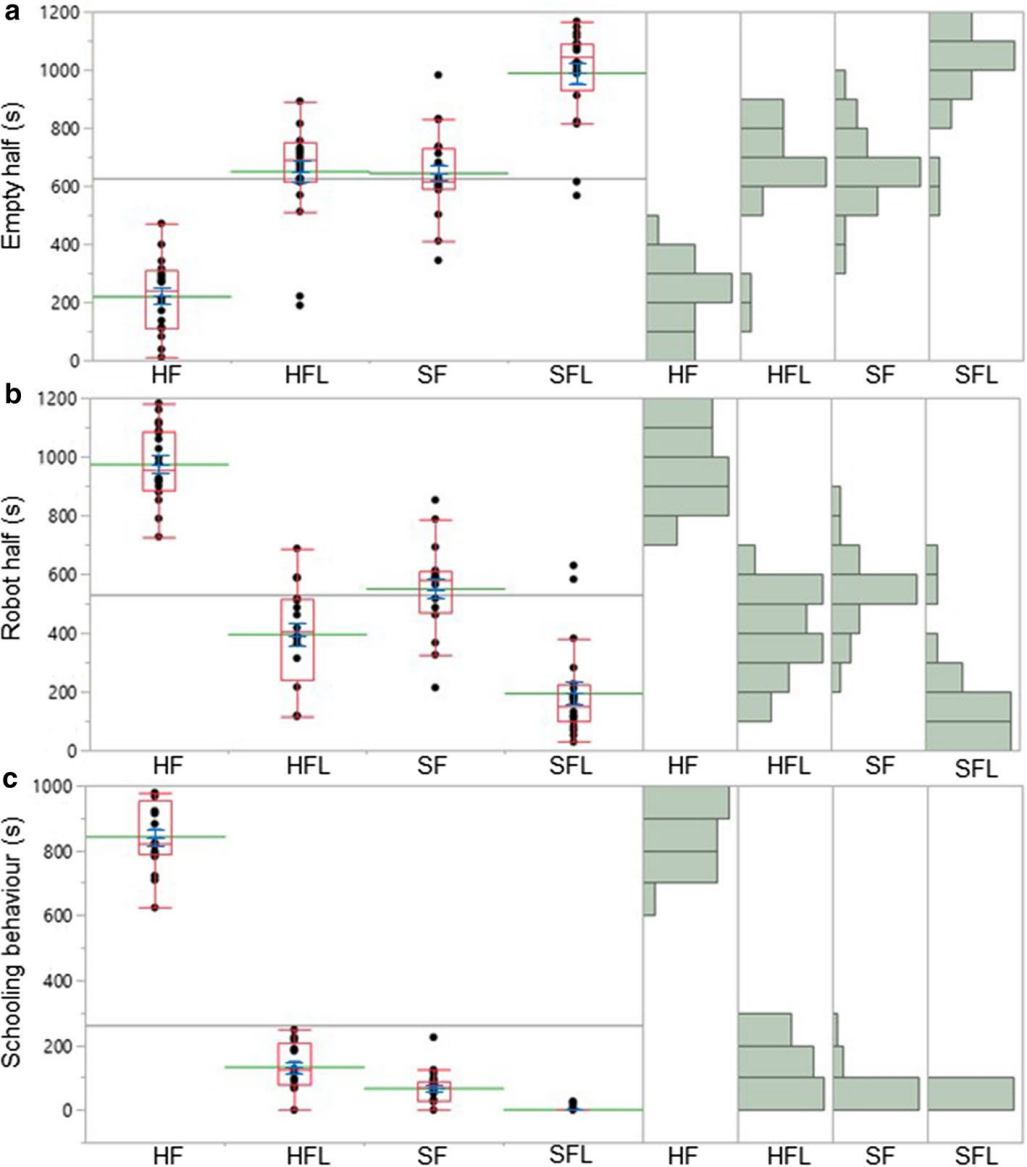
In *Paracheirodon innesi* subjects, the time spent in **a** the empty half, **b** the robot half, as well as **c** the schooling behaviour duration are affected by the healthy fish replica (HF), the healthy fish replica with locomotion anomalies (HFL), the sick fish replica (SF), the sick fish replica with locomotion anomalies (SFL) (Kruskal–Wallis test and Steel–Dwass test *P* > 0.05). The red line present in each box plot indicates the median and its range of dispersion (lower and upper quartiles, as well as outliers). The green line indicates the mean, and the blue T-bars show standard error value. For each box plot, on the right, data distribution is shown on histograms

The time spent in the empty half was significantly affected by different contexts (*χ*2 = 56.8; *d.f.* = 3; *P* < 0*.*0001). Fish spent a shorter time in the empty half in presence of the healthy fish replica compared to the sick fish replica with locomotion anomalies (*Z* = 5.396; *P* < 0.0001), the sick fish replica (*Z* = 5.315; *P* < 0.0001), the healthy fish replica with locomotion anomalies (*Z* = 4.801; *P* < 0.0001). Fish spent a longer time in the empty half in presence of the sick fish replica with locomotion anomalies compared to the sick fish (*Z* = 4.477; *P* < 0.0001), and to the healthy fish replica with locomotion anomalies (*Z* = 4.436; *P* < 0.0001) (Fig. 5a).

The time spent in the robot half was importantly affected by different contexts (*χ*2 = 59.6; *d.f.* = 3; *P* < 0*.*0001). Fish spent a longer time in the robot half in presence of the healthy fish replica compared to the healthy fish replica with locomotion anomalies (*Z* = −5.398; *P* < 0.0001), the sick fish replica (*Z* = −5.302; *P* < 0.0001), and to the sick fish replica with locomotion anomalies (*Z* = −5.397; *P* < 0.0001). Fish spent a longer time in the robot half in presence of the healthy fish replica with locomotion anomalies compared to the sick fish replica with locomotion anomalies (*Z* = −3.572; *P* = 0.0020).). Fish spent a longer time in the robot half in presence of the healthy fish replica with locomotion anomalies compared to the sick fish replica with locomotion anomalies (*Z* = −3.572; *P* = 0.0020). Fish spent a longer time in the robot half in presence of the sick fish replica compared to the healthy fish replica with locomotion anomalies (*Z* = −2.911; *P* = 0.0189). Furthermore, fish spent a longer time in the robot half in presence of the sick fish replica compared to the sick fish replica with locomotion anomalies (*Z* = −4.531; *P* < 0.0001) (Fig. 5b).

The schooling behaviour duration was significantly influenced by different contexts (*χ*2 = 62.3; *d.f.* = 3; *P* < 0*.*0001). Schooling behaviour was performed longer towards the healthy fish replica than towards the healthy fish replica with locomotion anomalies (*Z* = −5.399; *P* < 0.0001), the sick fish replica (*Z* = −5.401; *P* < 0.0001), and the sick fish replica with locomotion anomalies (*Z* = −5.617; *P* < 0.0001). Schooling behaviour lasted more when the healthy fish replica with locomotion anomalies was exposed, compared to when the sick fish replica (*Z* = −2.740; *P* = 0.0312), and the sick fish replica with locomotion anomalies (*Z* = −4.771; *P* < 0.0001), were exposed. Schooling behaviour was performed longer towards the sick fish replica than towards the sick fish replica with locomotion anomalies (*Z* = −4.460; *P* < 0.0001) (Fig. 5c).

## Discussion

Social species (including humans) are particular vulnerable to the transmission of diseases, due to high local population densities and prolonged interactions with conspecifics (Townsend et al. 2020). Social distancing, a behavioural-based response to diseases, has been reported to occur in nature, suggesting that this strategy provides benefits outweighing costs (Behringer et al. 2006; Mejía Salazar et al. 2016; Stroeymeyt et al. 2018; Townsend et al. 2020).

A challenging task for host species is to identify distinctive stimuli associated with an infectious disease. However, the study of behavioural avoidance in response to infection by pathogens and infestation by parasites in laboratory conditions is complex. Indeed, the experimental use of free-swimming real infected animals interacting with healthy individuals would not be ethically acceptable (ASAB/ABS 2014; Nakayama and Saijo 2013; Romano et al. 2018). Furthermore, confining real infected animals in transparent cages, or the use of videoplaybacks (D'eath 1998; Rowland 1999; Petrazzini et al. 2012), would decrease the naturalness of the interaction, resulting in uncertain results. Animal-robot interactions and ethorobotics allow the use of biomimetic agents providing the possibility to fully control artefacts that exhibit more realistic visual and physical conspecific’s appearance (Bonnet et al. 2018; Bierbach et al. 2020; Macrì et al. 2020; Romano et al. 2020a), and at the same time cancelling the risk of transmission of diseases.

In this study, for the first time, infected conspecific-mimicking robotic agents were used to investigate social distancing in a group living species. Particularly, it provided the evidence that the social fish *P. innesi* is especially vigilant in identifying possible infectious sickness stimuli. In general, group living animals overgeneralize cues that can be related with a disease. In fact, wrongly considering a sick subject as healthy (false negative) can produce more severe costs for the fitness than wrongly considering a healthy subject as sick (false positive) (Zylberberg et al. 2013; Townsend et al. 2020). In our case, *P. innesi* individuals avoided the fish replica that mimicked a conspecific with morphological abnormalities (e.g. decoloured areas on the body) resembling a symptom associated with *P. hyphessobryconis*, a microsporidian parasite infesting neon tetras and other fish (Aiello et al. 1998; Michel et al. 2002; Novotný and Dvořák 2006). An important hypothetical point to consider is that the different colour pattern of the fish replica could not produce a real active avoidance behaviour, but a lack of social attraction towards what is perceived as a heterospecific. However, mixed-species aggregations have been commonly reported in many shoaling fish species (Ward et al. 2002; Paijmans et al. 2019). In the isolation condition of our experiments, *P. innesi* would most likely have affiliated with a heterospecific. So, we believe that the avoiding behaviour observed towards the fish replica with morphological abnormalities is a robust evidence that decoloured areas on the body of the fish replica were perceived as a symptom of an infecting disease.

Furthermore, social distancing was also triggered when *P. innesi* were exposed to the fish replica with an intact appearance, but performing swimming anomalies, an additional symptom caused by *P. hyphessobryconis*. This confirms the highly effectiveness of visual cues in transmitting information on the risk of infection (Behringer et al. 2018), although in underwater environments visual cues perception is sometime hampered by the colour, depth, and turbidity of the water (Johannesen et al. 2012; Ranåker et al. 2012). Therefore, in nature the limited possibility to perceive visual cues is often compensated by chemosensory systems due to their greater range of perception (Brown et al. 2004; Derby and Sorensen 2008). However, how different visual cues act in conjunction on triggering social distancing in animals, is poorly understood. Herein, we carried out a further test to study the multimodal influence of two different visual cues (morphology and locomotion anomalies), both associated with a disease status, and cutting out the effect of olfactive cues. When both morphology and locomotion anomalies were presented in conjunction by the fish replica, the impact on social distancing was significantly stronger than when presented individually. This indicates how fish may have evolved particular neural pathways used to identify different visual cues, and to associate their simultaneous presence with a greater severity of the infection. Similarly, in *Poecilia reticulata* Peters, individuals have been reported to avoid cues from conspecifics (e.g. both visual and chemical) in the later stages of infection (when the transmission speed and the number of parasites transmitting are higher), and this behaviour is used to precisely track the transmission risk (Stephenson et al. 2018).

The findings obtained through this biohybrid approach prompt more attention on the role of behavioural-based strategies to avoid pathogen/parasite diffusion. Indeed, while the immune system seems to be closely related to different aspects of the host ecology (Ricklefs 1992; Kundu and Faulkes 2004; Lee et al. 2008), the high flexibility of animal behaviour may play a fundamental role to contrast the emergence of a novel pathogen (Zylberberg et al. 2013).

Further research will focus on the blended effect of visual robotic cues with synthesized disease-borne olfactory cues.

Results from this study can be used to optimize computational approaches modelling disease dynamics to more accurately assess the spread of endemic and emerging pathogens and/or parasites in humans and wildlife contexts (Dwyer et al. 2005; Heesterbeek et al. 2015; Bekiros and Kouloumpou 2020).
